# Correction: Drif et al. Anti-Inflammatory and Cancer-Preventive Potential of Chamomile (*Matricaria chamomilla* L.): A Comprehensive In Silico and In Vitro Study. *Biomedicines* 2024, *12*, 1484

**DOI:** 10.3390/biomedicines13071595

**Published:** 2025-06-30

**Authors:** Assia I. Drif, Rümeysa Yücer, Roxana Damiescu, Nadeen T. Ali, Tobias H. Abu Hagar, Bharati Avula, Ikhlas A. Khan, Thomas Efferth

**Affiliations:** 1 Department of Pharmaceutical Biology, Institute of Pharmaceutical and Biomedical Sciences, Johannes Gutenberg University, Staudinger Weg 5, 55128 Mainz, Germany; adrif@uni-mainz.de (A.I.D.); r.damiescu@uni-mainz.de (R.D.); neltayeb@unimainz.de (N.T.A.); habuhaga@students.uni-mainz.de (T.H.A.H.); 2 National Center for Natural Products Research (NCNPR), School of Pharmacy, University of Mississippi, Oxford, MS 38677, USA; bavula@olemiss.edu (B.A.); ikhan@olemiss.edu (I.A.K.)

## Text Correction

There was an error in the original publication [1]. A correction has been made to the Results section (Section 3.12. Cell Death Detection) in the first two paragraphs of page. The correct terms “late apoptotic/necrotic” and “primary necrosis” have been added. Additionally, the percentages of late apoptotic/necrotic cells treated with 72 h quercetin 4 × IC50 and with 72 h lupeol 4 × IC50 have been corrected. The corrected paragraph is as follows:

“We investigated the mode of cell death in CCRF-CEM cells upon treatment with lupeol and quercetin for 72 h. The FITC-conjugated annexin V/PI assay was used to distinguish between living, early apoptotic, late apoptotic/necrotic, and primary necrotic cells. Annexin V is usually detected in early and late apoptosis. However, PI staining detects cells in late apoptosis and necrosis. Figure 10 shows that both compounds significantly induced cell death compared to the negative control. Quercetin induced late apoptosis/necrosis in 58.70% of cells at 4 × IC50 (*p* = 0.002), while lupeol induced late apoptosis/necrosis in 73.80% of cells (*p* = 0.001).”

A correction has been also made in the Discussion section, paragraph 12. The correct term “late apoptosis/necrosis” has been corrected. The corrected paragraph is as follows:

“In this context, it was interesting that the cytotoxicity of both compounds in CCRF-CEM resulted in late apoptosis/necrosis. This might be a cell-type-specific effect, as quercetin and lupeol have been reported to induce either apoptosis or necrosis (or necroptosis) in different cell lines [119–124]. Because apoptosis is driven by the balance of pro- and anti-apoptotic proteins in specific signaling cascades, it is known that mutations in specific genes encoding these proteins confer resistance to apoptosis [125–128]. As a consequence, cytotoxic insults may overcome apoptosis resistance by other cell death modes [129].”

## Figure Legend

In the original publication [1], there was a mistake in the legends for Figure 10. The measurement of apoptosis has been added, the legends A/C to lupeol and B/D to quercetin have been changed, and the correct term “late apoptosis/necrosis” has been added. There was also a slight mistake in the gating. The correct legends appear below.

Additionally, Figure 11 in the original publication, which presented Annexin V/PI staining results on GFP-transfected U2OS cells, is no longer considered valid. It was subsequently recognized that GFP fluorescence interfered with Annexin V-FITC detection, leading to signal overlap and potentially incorrect interpretation of apoptosis data. As a result, Figure 11 has been removed from the article. The corrected version of the paper will no longer include this figure. This correction does not affect the scientific conclusions of the study. The main findings remain unchanged, including: The IC_50_ values for quercetin (89.6 µM) and lupeol (60 µM) in U2OS cells are relatively high, indicating moderate cytotoxicity. The apoptosis assay (Annexin V/PI) was less critical for this cell line. The cell cycle analysis clearly showed G_2_/M arrest, further confirming the reliability of the cytotoxicity findings.

The authors apologize for any inconvenience caused and appreciate the opportunity to make this correction.

“**Figure 10.** Detection of cell death in CCRF-CEM cells using flow cytometry and annexin-V/PI staining to measure apoptosis using a flow cytometer. (**A**,**B**) Cells treated with 0.25 × IC_50_, 0.5 × IC_50_, 1 × IC_50_, 2 × IC_50_, and 4 × IC_50_ of quercetin and lupeol, for 72 h. DMSO was used as negative control. (**A**) Cells treated with lupeol and (**B**) cells treated with quercetin. Q1 represents necrotic cells (−) annexin V/(+) PI; Q2 represents late apoptotic cells exhibiting annexin V (+)/PI (+); Q3 represents early apoptotic cells (+) annexin V/(−) PI; Q4 represents viable cells (−) annexin V/(−) PI. (**C**,**D**) Bar diagrams representing the percentages of cells in the different quadrants. (**C**) Effects of lupeol and (**D**) Effects of quercetin. The treatment of both compounds at increasing concentrations significantly enhanced the percentage of necrotic cells. *** *p* < 0.001, ** *p* < 0.01, and * *p* < 0.05 compared to the negative control using paired two-tailed t-test. The bar diagrams were created based on the calculation of the mean values ± SD of three independent experiments.”

“Figure 11 has been removed due to technical issues that rendered the data invalid. This correction does not affect the results or conclusions of the original article.”

## Error in Figure

In the original publication [1], there was a mistake in Figures 10 and 11 as published. The gating in Figure 10 has been correctly adjusted, and Figure 11 has been removed, as it was not critical to the main findings of the study; its removal does not impact the conclusions of the article.

The corrected version appears below.

**Figure 10:**
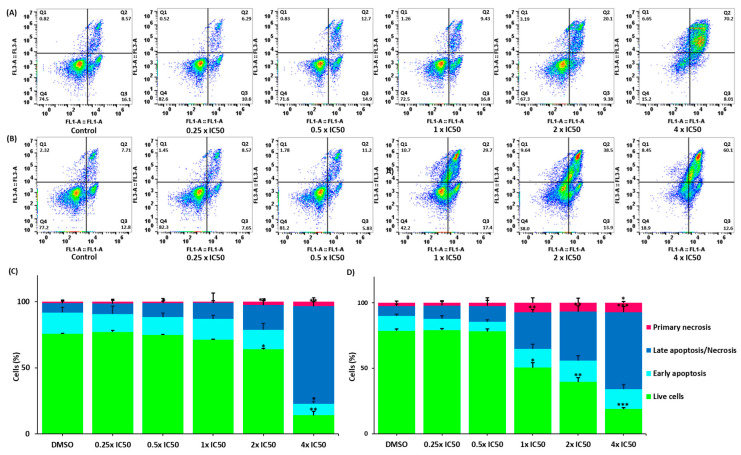
Detection of cell death in CCRF-CEM cells using flow cytometry and annexin-V/PI staining to measure apoptosis using a flow cytometer. (**A**,**B**) Cells treated with 0.25 × IC_50_, 0.5 × IC_50_, 1 × IC_50_, 2 × IC_50_, and 4 × IC_50_ of quercetin and lupeol, for 72 h. DMSO was used as negative control. (**A**) Cells treated with lupeol and (**B**) cells treated with quercetin. Q1 represents necrotic cells (−) annexin V/(+) PI; Q2 represents late apoptotic cells exhibiting annexin V (+)/PI (+); Q3 represents early apoptotic cells (+) annexin V/(−) PI; Q4 represents viable cells (−) annexin V/(−) PI. (**C**,**D**) Bar diagrams representing the percentages of cells in the different quadrants. (**C**) Effects of lupeol and (**D**) Effects of quercetin. The treatment of both compounds at increasing concentrations significantly enhanced the percentage of necrotic cells. *** *p* < 0.001, ** *p* < 0.01, and * *p* < 0.05 compared to the negative control using paired two-tailed t-test. The bar diagrams were created based on the calculation of the mean values ± SD of three independent experiments.

Figure 11 has been removed due to technical issues. This correction does not affect the results or conclusions of the original article. The authors state that the scientific conclusions are unaffected. This correction was approved by the Academic Editor. The original publication has also been updated.
