# Supplementary material for: Anti-Inflammatory and Cancer-Preventive Potential of Chamomile (Matricaria chamomilla L.): A Comprehensive In Silico and In Vitro Study
Source: Biomedicines. 2024 Jul 5;12(7):1484. doi: 10.3390/biomedicines12071484 (PMC11275008; doi:10.3390/biomedicines12071484)
Supplement: Supplementary file 1 [file biomedicines-12-01484-s001.zip › Supplementary Table S1.pdf]

Supplementary Table S1: Correlation of protein expression identified by COMPARE analysis with $log_{10}IC_{50}$ values for lupeol in the NCI panel of tumor cell lines.

No.	Symbol	Name	Cellular functions	Functional categories
1	ALDH4A1	Aldehyde dehydrogenase 4 family member A1	Proline degradation pathway by converting of pyroline-5-carboxylate to glutamate	Metabolism
2	ANXA1	Annexin A1	Inhibition of the phospholipase A2; anti-inflammatory activity	Immune function
3	AP1G1	Adaptor-related protein complex 1 subunit ɣ1	Intracellular protein trafficking between trans-Golgi network and endosomes	Protein trafficking
4	BCL2L13	BCL2-like 13	Maintaining mitochondrial integrity and regulating apoptosis	Cell death
5	CLIC4	Chloride intracellular channel 4	Stabilization of cell membrane potential, transepithelial transport, maintenance of intracellular pH, and regulation of cell volume	Ion channel
6	DBN1	Drebrin 1	Cytoplasmic actin-binding protein involved in neuronal growth	Cell proliferation and differentiation
7	ECH1	Enoyl-CoA hydratase 1	Fatty acid β-oxidation	General metabolism
8	EIF5	Eukaryotic translation initiation factor 5	GTP hydrolysis, forms the 80S ribosomal initiation complex	DNA/RNA metabolism
9	EPCAM	Epithelial cell adhesion molecule	Cell adhesion molecule; Antigen expressed on most normal epithelial cells and gastrointestinal carcinomas	Cell adhesion
10	ERP44	Endoplasmic reticulum protein 44	Chaperone in the secretory pathway; protein quality control at the endoplasmic reticulum-Golgi interface	Chaperones
11	FHL1	Four and a half LIM domains 1	Zinc-finger protein involved in many cellular processes	Transcription factor
12	FLNC	Filamin C	Crosslinking actin filaments into networks and anchoring membrane proteins to the actin cytoskeleton	Cytoskeleton
13	FSTL1	Follistatin-like 1	Activin-binding protein; regulation of immune response	Immune function
14	HOMER3	Homer scaffold protein 3	Binds glutamate receptors, inositol 1,4,5-trisphosphate receptors, and amyloid precursor proteins, influencing neuronal signaling, T-cell activation, and amyloid beta peptide trafficking	Signal transduction; immune function
15	HSD17B12	Hydroxysteroid 17-β dehydrogenase 12	Fatty acid elongation. Estrone into estradiol convertion in ovarian tissue	General metabolism

16	LGALS3	Lectin, galactoside-binding, soluble, 3	Binds to β-galactoside-containing glycoconjugates. Involved in apoptosis, innate immunity, cell adhesion and T-cell regulation	Cell death; immune function; cell adhesion
17	LGALS3BP	Lectin galactoside-binding soluble 3-binding protein	Modulation cell-cell and cell-matrix interactions. Regulating immune responses associated with natural killer and lymphokine-activated killer	Immune function
18	LMAN2	Lectin, mannose-binding 2	Shuttles between the endoplasmic reticulum, the Golgi apparatus and the plasma membrane. Binds high mannose type glycoproteins and may facilitate their sorting, trafficking, and quality control	Protein trafficking
19	MGST3	Microsomal glutathione S-transferase 3	Inflammation mediator through the production of leukotrienes and prostaglandin E	Immune function
20	MLKL	Mixed lineage kinase domain-like pseudokinase	Inhibits tumor necrosis factor (TNF)-induced necroptosis (its inhibition prevents TNF-induced necrosis)	Cell death
21	MSN	Moesin (membrane-organizing extension spike protein)	Cross-linker between plasma membranes and the actin-based cytoskeleton	Cytoskeleton
22	NAP1L1	Nucleosome assembly protein 1-like 1	DNA replication and modulator of chromatin formation	DNA/RNA metabolism
23	PDAP1	PDGFA-associated protein 1	modulates fibroblast growth by regulating platelet-derived growth factors A and B	Cell proliferation and differentiation
24	PFDN1	Prefoldin subunit 1	Chaperone complex that stabilizes newly synthesized polypeptides	Chaperones
25	PLIN3	Perilipin 3	Transport of lysosomal hydrolase from endosomes to the Golgi complex	Protein trafficking
26	PRPF38B	Pre-mRNA processing factor 38B	Involved in mRNA splicing through the splicesome pathway	DNA/RNA metabolism
27	PRPS1	Phosphoribosyl pyrophosphate synthetase 1	Phosphoribosylation of ribose 5-phosphate to produce 5-phosphoribosyl-1-pyrophosphate (PRPP)	DNA/RNA metabolism
28	RAB5B	Ras-related protein Rab-5B	GTPase activity; antigen-processing and presentation; transport from lasma membrane to endosome	Protein trafficking; immune function
29	RABL3	Rab-Like Protein 3	GTPase activity, Ras signal transduction reguation	Signal transduction
30	SEC62	SEC62 homolog, preprotein translocation factor	Post-translational protein translocation into the endoplasmic reticulum and backward transport of ER proteins to the ubiquitin-proteasome-dependent degradation pathway	Protein trafficking
31	SLC25A1	Solute carrier family 25 member 1	Translocates small metabolites across the mitochondrial membrane	Transporter
32	SLC30A1	Solute carrier family 30 member 1	Calcium channel inhibitor and zinc ion transmembrane transporter	Transporter

33	STAU1	Staufen double-stranded RNA binding protein 1	mRNA transport via the microtubule network to the RER for translation; binds to microtubules	DNA/RNA metabolism; cytoskeleton
34	SUV39H2	Suppressor of variegation 3-9 homolog 2/ SUV39H2 histone lysine methyltransferase	Chromatin assembly/disassembly and remodeling. Role in cellular responses to hypoxia and regulation of transcription by RNA polymerase II	DNA/RNA metabolism
35	TAGLN	Transgelin	Calcium-independent smooth muscle contractions; tumor supressor activity	Tumor suppressor
36	TECR	Trans-2,3-enoyl-CoA reductase	Elongation of microsomal long and very long chain fatty acids in the endoplasmic reticulum	General metabolism
37	TLN1	Talin 1	Attachment of adherent cells to extracellular matrices and facilitating lymphocyte adhesion to other cells	Cell adhesion, immune function
38	TPM4	Tropomyosin 4	Involved in the contractile system of striated and smooth muscles and the cytoskeleton of non-muscle cells; provides stability to the filaments and regulate access of other actin-binding proteins	Cytoskeleton
39	VAPB	VAMP (vesicle-associated membrane protein)-associated protein B and C	Forms homodimers and heterodimers with VAPA, and interacts with VAMP1 and VAMP2	Vesicle trafficking
40	YWHAZ	Tyrosine 3-monooxygenase/tryptophan 5-monooxygenase activation protein ζ	Signal transduction by binding to phosphoserine-containing proteins	Signal transduction
