# Supplementary material for: Anti-Inflammatory and Cancer-Preventive Potential of Chamomile (Matricaria chamomilla L.): A Comprehensive In Silico and In Vitro Study
Source: Biomedicines. 2024 Jul 5;12(7):1484. doi: 10.3390/biomedicines12071484 (PMC11275008; doi:10.3390/biomedicines12071484)
Supplement: Supplementary file 1 [file biomedicines-12-01484-s001.zip › Supplementary Table S2.pdf]

Supplementary Table S2: Correlation of protein expression identified by COMPARE analysis with $\log_{10}IC_{50}$ values for quercetin in the NCI panel of tumor cell lines.

No.	Symbol	Name	Cellular functions	Functional categories
1	STX7	Syntaxin 7	Mediates the coordinated fusion of endosomes and lysosomes with the phagosome	Vesicle trafficking
2	CAPG	Capping actin protein, gelsolin-like	Regulates actin dynamics and actin-based motility in non-muscle cells	Cytoskeleton
3	CHCHD3	Coiled-coil-helix-coiled-coil-helix domain-containing 3	Component of the mitochondrial contact site and cristae organizing system (MICOS). Regulation of ATP production, cell growth, and decreased oxygen consumption	Mitochondrial function
4	MFN1	Mitofusin 1	Mitochondrial membrane protein facilitating mitochondrial targeting	Mitochondrial function
5	MLANA	Melan-A	Melanin biosynthesis in the endoplasmic reticulum membrane, melanosome, and trans-Golgi network	Melanosome biosynthesis and function
6	MTX2	Metaxin 2	Protein import into the mitochondrion	Mitochondrial function
7	SAMM50	Sorting and assembly machinery component 50	Assembling β-barrel proteins into the outer mitochondrial membrane	Mitochondrial function
8	ZC3H11A	Zinc finger CCCH-type-containing 11A	RNA binding activity; export of poly(A)+ mRNA from the nucleus	DNA/RNA metabolism
9	PMEL	Premelanosome protein	Structural organization of premelanosomes, melanoma-specific serum marker	Melanosome biogenesis and function
10	CHCHD6	Coiled-coil-helix-coiled-coil-helix domain-containing 6	Cellular response to DNA damage and cristae formation; located in the cytosol and mitochondrial inner membrane; part of the MICOS complex	Mitochondrial function; DNA damage response
11	RAB32	RAB32, member RAS oncogene family	Facilitates mitochondrial fission; involved in autophagy and melanosome secretion	Mitochondrial function; cell death; melanosome bio-genesis and function
12	GAPDHS	Glyceraldehyde-3-phosphate dehydrogenase, spermatogenic	Carbohydrate metabolism; regulates energy pathways during spermiogenesis, impacting sperm motility and male fertility	General metabolism
13	ACOT7	Acyl-CoA thioesterase 7	Hydrolyzes the CoA thioester of palmitoyl-CoA and other long-chain fatty acids	General metabolism
14	RALY	RALY heterogeneous nuclear ribonucleoprotein	Pre-mRNA splicing and embryonic development	DNA/RNA metabolism
15	VAMP3	Vesicle-associated membrane protein 3	Docking and/or fusion of synaptic vesicles with the presynaptic membrane	Synaptic transmission

16	ACO2	Aconitase 2	Conversion of citrate to isocitrate via cis-aconitate in the TCA cycle; Involved in the energy metabolim in mitochondria	General metabolism; mitochondrial function
17	HEXA	Hexosaminidase subunit α	Generation of the α subunit of the lysosomal enzyme β-hexosaminidase; degradation of the ganglioside GM2	Lysosomal function
18	GGH	ɣ-Glutamyl hydrolase	Hydrolysis of folylpoly-γ-glutamates and antifolylpoly-ɣ glutamates	General metabolism
19	VAPB	VAMP (vesicle-associated membrane protein)-associated protein B/C	Located in plasma and intracellular vesicle membrane; vesicle trafficking	Vesicle trafficking
20	MCAM	Melanoma cell adhesion molecule	Glomerular filtration and vascular wound healin; functions upstream or within angiogenesis	Cell adhesion
21	COPS4	COP9 signalosome subunit 4	Positive regulator of SCF-type E3 ubiquitin ligases	Signal transduction
22	YARS	Tyrosyl-tRNA synthetase	Aminoacylation of tRNA with tyrosine; exhibits cytokine activities	Immune function
23	CCS	Copper chaperone for superoxide dismutase	Delivers copper to copper/zinc superoxide dismutase and activates it	Chaperone
24	TRIP13	Thyroid hormone receptor interactor 13	Interacts with thyroid hormone receptors; involved in non-small cell lung cancer; role in chromosome recombination and chromosome structure development during meiosis	DNA/RNA metabolism
25	DYNC1LI1	Dynein cytoplasmic 1 light intermediate chain 1	Intracellular trafficking and chromosome segregation during mitosis; role in moving the spindle assembly checkpoint (SAC) from kinetochores to spindle poles; mediates binding to other cargo molecules to facilitate intracellular vesicle trafficking	Vesicle trafficking; DNA/RNA metabolism
26	MRPS30	Mitochondrial ribosomal protein S30	Protein synthesis within the mitochondrion	Mitochondrial function
27	COPG1	COPI coat complex subunit ɣ1	Golgi vesicle transport and organelle transport along microtubules	Vesicle trafficking
28	EXOSC1	Exosome component 1	Degradation of AU rich element-containing RNAs	DNA/RNA metabolism
29	PPP5C	Protein phosphatase 5 catalytic subunit	Regulation of reversible protein phosphorylation at serine and threonine residues, regulation of cell growth and differentiation through different signaling pathways	signal transduction; cell proliferation and differentiation
30	RQCD1	CCR4-NOT transcription complex subunit 9	Signal transduction and retinoic acid-regulated cell differentiation	Signal transduction; cell proliferation and differentiation
31	ATP5F1	ATP synthase peripheral stalk-membrane subunit B	ATP synthesis during oxidative phosphorylation	Mitochondrial function
32	DNM2	Dynamin 2	Involved in endocytosis and cell motility; binds proteins that bind actin and cytoskeletal proteins; GTPase activity	Cytoskeleton

33	EIF3J	Eukaryotic translation initiation factor 3 subunit J	Initiation of translation through recruitment of protein and mRNA to the 40s ribosome	DNA/RNA metabolism
34	PFAS	Phosphoribosylformyl-glycinamidine synthase	Catalyzes fourth step of inositol-monophosphate biosynthesis	DNA/RNA metabolism
35	UBQLN2	Ubiquilin 2	Links the ubiquitination machinery to the proteasome	Protein degradation
36	PDCD5	Programmed cell death 5	Regulator of lysine acetyltransferase 5; involved in DNA transcription, DNA repair, and cell cycle control	Cell death; DNA/RNA metabolism; cell proliferation and differentiation
37	PRPF38A	Pre-mRNA processing factor 38A	mRNA splicing in the nucleoplasm	DNA/RNA metabolism
38	CAPN1	Calpain 1	Proteolysis of substrates involved in cytoskeletal remodeling and signal transduction	Cytoskeleton; signal transduction
39	POR	Cytochrome P450 oxidoreductase	Metabolism of steroid hormones, drugs, and xenobiotics	General metabolism
40	SYAP1	Synapse-associated protein 1	Regulates TORC2 signaling; cellular response to growth factor and peptide hormone stimuli	Signal transduction
