# Supplementary figures and images for: Anti-Inflammatory and Cancer-Preventive Potential of Chamomile (Matricaria chamomilla L.): A Comprehensive In Silico and In Vitro Study
Source: Biomedicines. 2024 Jul 5;12(7):1484. doi: 10.3390/biomedicines12071484 (PMC11275008; doi:10.3390/biomedicines12071484)

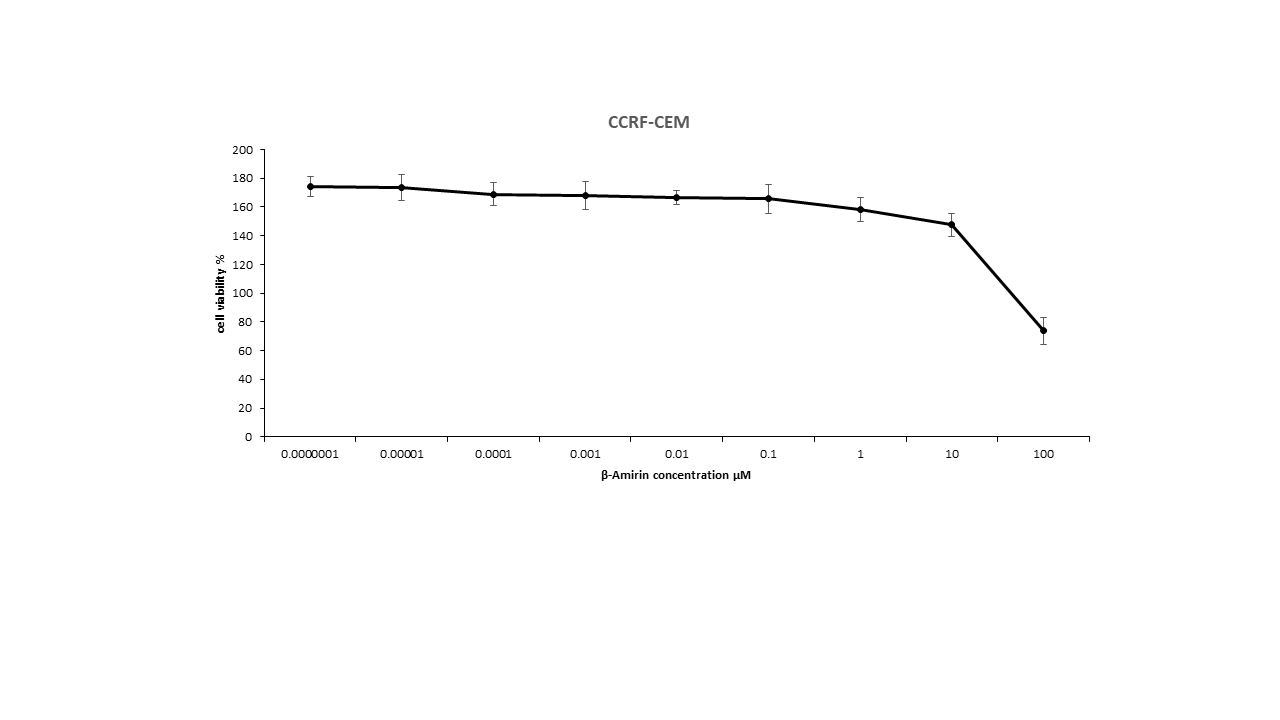

Supplement: Supplementary file 1 [file biomedicines-12-01484-s001.zip › Supplementary Figure S1.tif]
